# Shear Wave Dispersion Elastography in ALD and MASLD: Comparative Pathophysiology and Clinical Potential—A Narrative Review

**DOI:** 10.3390/jcm13247799

**Published:** 2024-12-20

**Authors:** Tommaso Dionisi, Linda Galasso, Luigiandrea Antuofermo, Francesco Antonio Mancarella, Giorgio Esposto, Irene Mignini, Maria Elena Ainora, Antonio Gasbarrini, Giovanni Addolorato, Maria Assunta Zocco

**Affiliations:** 1 Unit of Internal Medicine, Department of Medical and Surgical Sciences, IRCCS “A. Gemelli” University Polyclinic Foundation, 00168 Rome, Italy; tommaso.dionisi1@guest.policlinicogemelli.it (T.D.); francescoantonio.mancarella@policlinicogemelli.it (F.A.M.); antonio.gasbarrini@policlinicogemelli.it (A.G.); giovanni.addolorato@policlinicogemelli.it (G.A.); 2 Internal Medicine and Alcohol Related Disease Unit, Columbus-Gemelli Hospital, Fondazione Policlinico Universitario A. Gemelli IRCCS, 00168 Rome, Italy; luigiandrea.antuofermo01@icatt.it; 3 Department of Internal Medicine and Gastroenterology, Fondazione Policlinico Universitario Agostino, Gemelli IRCCS, Catholic University of Rome, 00168 Rome, Italy; linda.galasso@guest.policlinicogemelli.it (L.G.); giorgio.esposto@guest.policlinicogemelli.it (G.E.); irene.mignini@guest.policlinicogemelli.it (I.M.); mariaelena.ainora@policlinicogemelli.it (M.E.A.); 4 CEMAD Digestive Disease Center, Fondazione Policlinico Universitario Agostino, Gemelli IRCCS, Catholic University of Rome, 00168 Rome, Italy

**Keywords:** ALD, SWD, elastography, biomarker, fibrosis, necroinflammation, liver disease

## Abstract

Alcohol-related liver disease (ALD) is a major cause of global morbidity and mortality, progressing from steatosis to cirrhosis and hepatocellular carcinoma. While liver biopsy remains the gold standard for identifying liver disease, non-invasive methods like shear wave dispersion (SWD) elastography offer promising alternatives. This scoping review evaluates SWD’s potential in the study of ALD, comparing it to metabolic dysfunction-associated steatotic liver disease (MASLD). SWD measures changes in shear wave speed in relation to liver viscosity and necroinflammation. Studies in MASLD suggest that SWD effectively correlates with fibrosis and inflammation stages, but its application in ALD remains underexplored. Both ALD and MASLD show similar inflammatory and fibrotic pathways, despite having different etiologies and histological features. This review emphasizes the necessity to identify ALD-specific SWD reference values and verify SWD’s ability to improve diagnosis and disease progression. Prospective studies comparing SWD findings with histological benchmarks in ALD are essential for establishing its clinical utility. Incorporating SWD into clinical practice could revolutionize the non-invasive evaluation of ALD, offering a safer, cost-effective, and repeatable diagnostic tool.

## 1. Introduction

According to the World Health Organization, alcohol use results in 3.3 million deaths each year, representing 6% of all global deaths, and is implicated in nearly half of cirrhosis cases [1]. AUD affects approximately one in twelve adults, meeting at least two out of the eleven DSM-V diagnostic criteria, along with a consumption of over three drinks per day for males and over two drinks per day for females. Only 10–20% of chronic heavy drinkers experience severe conditions such as alcoholic hepatitis or cirrhosis [1]. However, alcoholic liver disease (ALD) accounts for a large part of chronic liver disease (CLD) globally, playing a significant role in preventable illnesses and liver-related death rates internationally.

ALD encompasses a continuum of liver diseases from alcohol-related steatosis to steatohepatitis (ASH), and it extends to fibrosis or cirrhosis [2]. All the grades are defined histologically, with cirrhosis progression often occurring without symptoms, which complicates early diagnosis. About 90–100% of individuals consuming over 40 g of alcohol daily for years will develop fatty liver disease [3,4]; among these, 10–35% advance to ASH, marked by liver inflammation and fibrogenesis.

Liver fibrosis, stemming from repeated inflammation and injury, can lead to cirrhosis. Moreover, 8–20% of those with ASH might progress to cirrhosis, increasing the risk of hepatocellular carcinoma in approximately 2% of cases [3,4]. Sustained alcohol abstinence is the only effective treatment for halting the progression of ALD and potentially reversing liver damage.

Diagnosing and assessing liver damage in ALD relies on both invasive and non-invasive methods. While liver biopsy is the gold standard for evaluating steatosis, inflammation, and fibrosis, its invasiveness limits routine use. Non-invasive alternatives, such as transient elastography (TE), magnetic resonance elastography (MRE), and MRI with proton density fat fraction (MRI-PDFF), are effective for assessing liver stiffness and fat content [5,6]. Additionally, ultrasound-based techniques like attenuation imaging and shear wave elastography (SWE) are widely adopted in clinical practice.

Reduced immune function and intestinal bacterial translocation put ALD patients at risk of infections and sepsis. Sepsis risk increases necroinflammation, worsening ALD treatment and prognosis. Shear wave dispersion (SWD) elastography can detect tissue viscosity to monitor necroinflammation and suggest more prompt treatment interventions [7]. Identifying necroinflammation and advanced fibrosis is vital in ALD for thwarting liver-related events. Transient elastography (TE) and shear wave elastography (SWE) measure liver stiffness by quantifying shear wave speed and tissue density. Shear waves move faster in stiffer tissue when they are caused by outside pressure, transducer vibration, or ARFI, reflecting the histological characteristics of liver parenchyma [6].

Despite these advancements, accurately assessing liver inflammation, particularly in ALD, remains challenging. SWD offers a promising solution by evaluating tissue viscosity alongside elasticity, providing indirect insights into necroinflammatory activity [8,9,10]. This technique captures dynamic changes in liver tissue, making it a potential tool for addressing the diagnostic gap in ALD and complementing existing non-invasive methods for assessing inflammation, fibrosis, and steatosis [11].

SWD assesses changes in shear wave velocity based on frequency and in relation to liver viscosity and necroinflammation. In metabolic dysfunction-associated steatotic liver disease (MASLD), SWD is linked to the stages of fibrosis and inflammation severity [8,9,10]. SWD also aids in evaluating liver transplant allograft deterioration, indicating it can identify necroinflammation.

Sun et al.’s study found that SWD imaging is a valuable non-invasive metric for assessing liver disease complications, including variceal hemorrhage, in cirrhotic patients [11]. Nonetheless, no research has yet examined SWD in the context of alcoholic liver diseases [9,10,11,12].

Recent advancements in SWD have expanded its applications beyond traditional liver diseases, including emerging contexts such as myeloproliferative neoplasms, which offer insights into its broader diagnostic potential and highlight the need for further exploration in conditions like ALD [13].

This scoping review explores the potential of SWD as a diagnostic tool for ALD.

Studies were identified through a systematic search approach of PubMed using keywords such as ‘shear wave elastography’, ‘liver stiffness’, ‘ALD’, and ‘MASLD’. Only peer-reviewed studies published in English were included.

This review compares ALD and metabolic-associated fatty liver disease (MAFLD), emphasizing the shared diagnostic challenges and insights from SWD research in MAFLD. It assesses SWD’s ability to evaluate liver steatosis, necroinflammation, and fibrosis, identifies knowledge gaps, and outlines future research directions to establish SWD as a non-invasive, reliable, and clinically valuable diagnostic tool for ALD.

## 2. Comparison of ALD and MASLD

ALD and MASLD have different etiologies but a similar course concerning the evolution from steatosis to the most severe stages of fibrosis and cirrhosis [14,15]. Understanding both the molecular and histological pathogeneses of these conditions is essential for extending the indications of diagnostic approaches and therapeutic targets from one pathology to another.

Ethanol metabolism primarily triggers ALD through enzymes such as alcohol dehydrogenase (ADH) and cytochrome P450 2E1 (CYP2E1), increasing acetaldehyde and NADH. This modification impairs fat metabolism and can lead to hepatic steatosis, the first pathological stage of ALD [16]. Conversely, MASLD is driven by insulin resistance, which leads to increased lipolysis in adipose tissue, elevated free fatty acid levels in the liver, and enhanced triglyceride synthesis and accumulation, thus initiating steatosis [17].

Histologically, steatosis in both MASLD and ALD involves the accumulation of fat in hepatocytes, but with different patterns of lipid storage. As for the first, fat accumulates as large droplets (macrovesicular steatosis) that push the nucleus to the periphery of the cell [18]. In alcoholic steatosis, along with macrovesicles, small fat droplets accumulate without displacing the nucleus (microvesicular steatosis), reflecting disruptions in lipid metabolism due to alcohol metabolites [19].

Both diseases promote steatosis via different mechanisms—for example, excess NADH and acetaldehyde upregulate de novo lipogenesis and inhibit the secretion of very-low-density lipoproteins, trapping lipids in the hepatocytes in ALD, [14] while in MASLD, hepatic uptake of fatty acids for conversion into triglycerides is upregulated. Insulin resistance, on the other hand, impairs beta-oxidation, further worsening fat accumulation [20]. Chronic alcohol intake and a high-fat diet can overlap in their effects on liver metabolism and the gut–liver axis. Though ALD and MASLD are considered distinct, their mechanisms intersect. Moderate alcohol may temporarily reduce high-fat diet effects, but oxidative stress and microbiome changes worsen liver damage, linking these conditions [21]. Despite these differences, both cause hepatocyte lipid accumulation and liver disease progression.

Oxidative stress contributes to cellular damage in both ALD and MASLD. In ALD, ethanol metabolism by the MEOS system increases ROS [22], the peroxidation of excess fatty acids, while in MASLD, the result is oxidative damage to lipids, proteins, and DNA itself, affecting cellular functions and finally resulting in cell death by apoptosis or necrosis [23]. In this oxidizing environment, there is an increase in inflammatory pathways, leading to the upregulation of NF-κB. As a result, metabolic dysregulation occurs, which is associated with inflammatory responses [24].

Inflammatory triggers show clear differences. In ALD, the primary driver of inflammation is acetaldehyde toxicity in association with oxidative stress, stimulating Kupffer cells to release pro-inflammatory cytokines such as TNF-α, IL-1β, and IL-6.

On the other hand, in MASLD, fatty acids and their by-products significantly drive inflammation through toll-like receptors and other innate immune system constituents. Both these disorders witness these cytokines not merely inflicting hepatocyte damage themselves but also recruiting more immune cells into the liver, thus extending the inflammatory cascade [25,26]. In MASLD, particularly in MASH, inflammation is defined by lymphocyte and macrophage penetration alongside hepatocyte ballooning [27]. In ALD though, neutrophils are notably active near hepatocytes marked by Mallory–Denk bodies—indicative features of liver damage predominantly linked with ALD [28]. Each disorder involves the release of damage-associated molecular patterns (DAMPs) from injured hepatocytes and pathogen-associated molecular patterns (PAMPs), which further stimulate immune responses [29].

Despite these different initial triggers, the inflammatory pathways significantly overlap. Both conditions involve similar cytokines and immune cell recruitment. Cross-talk occurs among damaged hepatocytes, immune cells, and activated hepatic stellate cells (HSCs), triggering a positive feedback loop that promotes inflammation and fibrosis in both diseases [30].

Fibrosis patterns can help differentiate between MASLD and ALD. MASLD-related fibrosis generally starts in the periportal areas, forming bridging fibrosis in a ‘chicken wire’ pattern [31], whereas, ALD fibrosis begins around the central veins and extends pericellularly [32]. Unique to ALD are Mallory–Denk bodies, which are linked to cell death, and megamitochondria, a result of chronic alcohol exposure, highlighting the mitochondrial stress and altered bioenergetics specific to alcohol-induced liver damage [33,34].

Significant differences in liver stiffness measurements (LSMs) are observed between MASLD and ALD. Non-invasive methods such as transient elastography (TE) revealed that ALD generally produces higher LSM values compared to MASLD, particularly in advanced disease stages. For MASLD, advanced fibrosis is typically indicated by TE values around 7.6–8.0 kPa, with cirrhosis exceeding 10.3–11.3 kPa. In contrast, ALD demonstrated significant fibrosis at TE values of 6.9–11.6 kPa and cirrhosis ranging from 12.1 to 25.9 kPa [35].

Systemic interactions play a role in both diseases, especially through the gut–liver and adipose–liver connections.

In ALD, problems with the gut barrier let more endotoxins in, causing inflammation. MASLD faces similar issues due to diet-related changes in gut bacteria [36,37].

Another connection between systemic metabolic health and liver disease is the adipose–liver axis, which is influenced by altered adipokine production in ALD and increased free fatty acid release in MASLD [38,39].

Systematic analyses of molecular pathways have found shared mechanisms, like cytokine-driven inflammation, and unique aspects of ALD, such as specific metabolic alcohol-related alteration compared to insulin resistance in MASLD.

## 3. SWD: Principles and Clinical Implementation

The novel ultrasonic technology, shear wave dispersion (SWD), is based on shear wave elastography. It measures the changes in shear wave (SW) speeds, adjusting for the frequency. This method is quite efficient in evaluating liver tissue, distinguishing healthy livers from steatosis and cirrhosis [10,40,41].

Current software can show SWD as the shear wave dispersion slope (SWDS), quantified in (m/s)/kHz [42,43,44,45], or as a viscosity index, denoted in Pa·s [46,47]. These measurements help detect liver diseases related to liver viscosity [43,48] and track their progression [49,50]. This progression is seen as increased liver stiffness [51] and shear wave propagation patterns within the liver [52,53,54].

### 3.1. Advantages and Challenges of SWD

There are numerous advantages for the SWD technique. It eliminates the need for radiation, ensuring patient safety. It is inexpensive and widely accessible compared to CT and MRI, allowing repeatable and real-time evaluations. SWD offers impartial analysis, reducing the variability in a typical ultrasound operator in B mode. Conversely, the technology poses challenges that need to be addressed. SWD is operator-based, may have penetration problems in obese or ascites patients, and shows variability in different ultrasound systems. Standardization and subsequent validation of SWD are required in various patient groups. CT and MRI usually provide a higher spatial resolution, resulting in more detailed anatomical information than SWD techniques.

### 3.2. SWD Reference Values in Patients with Liver Diseases

Healthy and fibrotic livers can be identified by their SWD scores.

Trout et al. (2020) reported mean SWDS values of 11.43 ± 1.75 (m/s)/kHz in children and 10.24 ± 1.65 (m/s)/kHz in adults, suggesting slight age-related differences in liver viscoelasticity [55]. Similarly, Sugimoto et al. (2020) observed a mean viscosity of 1.59 Pa·s in healthy adults, with significant correlations between viscosity, BMI, and age. These findings emphasize the necessity of stratifying SWD reference values based on demographic factors [9].

Age-related viscoelasticity changes may explain the different SWD values in pediatric and adult livers [55,56]. Increasing collagen and liver stiffness (LS) increase with age [55,57]. Thus, age stratification is essential for appropriate diagnostic score interpretation. The effects of BMI [55,58] and wall thickness [55] on these measurements are unknown.

In a prospective investigation of adult cirrhotic patients, Deffieux et al. [59] measured SWD using the viscosity index and found higher viscosity levels reflecting hepatic parenchymal inflammation.

Multiple studies have examined this technique in different populations. SWD is measured using SWDS [60,61], applied in patients with MASH [61], alpha-1 antitrypsin deficiency [62], liver transplantation [12], and advanced chronic liver disease (CLD) of mixed etiology [14]. According to our knowledge, there is a lack of research on alcohol-induced liver disease.

### 3.3. Clinical Protocol for SWD in ALD

The SWD process requires a patient’s preparation, including an explanation of the purpose and the outcome. If applicable to SWD, fasting should be followed for best results. During the procedure, the patient should be positioned comfortably, usually lying down. The measurements require an ultrasound device that allows the screen to be divided, the image to be frozen, and the possibility to set the region of interest (ROI) for multiple measurements, resulting in an average result.

## 4. SWD and Study of the Liver

The molecular as well as histological alteration that underlie shifts in liver viscoelasticity have become a hot topic in liver disease research. Inflammation, fibrosis, and steatosis are key factors that affect liver tissue viscosity, and they may undergo evaluation via SWD imaging [63]. SWD can identify microstructural changes, providing perspective in the modification occurring at a molecular level [64].

### 4.1. Steatosis

Liver steatosis is characterized by augmented triglyceride content in the hepatocytes, significantly modifying liver viscosity and thereby impacting SWD readings. Generally, the correlation of SWD with steatosis alone is weaker than its correlation with fibrosis or inflammation. Nevertheless, SWD may distinguish steatosis and inflammation histological grades.

In a prospective study, Wang et al. (2022) examined the impact of SWD on hepatic steatosis. SWE and SWD were conducted on 210 HCC patients scheduled for hepatectomy. The fibrosis stage and necroinflammatory activity were assessed by biopsy. The findings indicated an absence of a notable correlation between hepatic stiffness and steatosis, emphasizing the intricacies of liver tissue attributes. Nonetheless, triglyceride collection within hepatic cells impacts liver viscosity, thereby altering SWD assessments. This finding underscores the importance of recognizing changes in liver viscoelasticity while interpreting SWD data for assessing steatosis [51].

Research studies have shown a weaker ability of SWD to distinguish different histological grades of steatosis. A study by Ferraioli et al. used transient elastography (TE) as a standard to show that 2D-SWE was more accurate at measuring liver fibrosis but not steatosis in people with chronic liver disease [65]. At the same time, Deffieux et al. indicated a relationship between viscosity metrics and fibrosis, yet no such association with steatosis or disease activity manifests in CLD-afflicted individuals. This underscores the challenge presented when attempting to quantify steatosis using solely the SWD methodology [59]. In a pediatric population with CLD, research by Cetinic et al. suggested that attenuation imaging (ATI) exhibits a greater correlation with steatosis than SWD [56].

Similarly, Schulz et al. found that both the ATI and the controlled attenuation parameter (CAP) yield elevated steatosis measurements in subjects deficient in alpha-1 antitrypsin (AATD) [66].

#### Steatosis in MASLD

Different ultrasound-based techniques have demonstrated utility in assessing steatosis in MASLD. Popa et al. (2021) sought to evaluate the performance of SWD in assessing hepatic steatosis in MASLD patients via a cross-sectional study of 215 adults with MASLD who underwent MPUS examinations using four techniques: 2D-SWE.PLUS, Sound Speed Plane-Wave UltraSound (SSp.PLUS), Attenuation Plane-Wave UltraSound (Att.PLUS), and Viscosity Plane-Wave UltraSound (Vi.PLUS). TE with CAP served as the reference method. For steatosis assessment, SSp.PLUS correlated better with CAP values compared to Att.PLUS. The optimal SSp.PLUS cut-off value for significant steatosis (S ≥ 2) was identified [67]. Sugimoto et al. (2021) confirmed these results by further investigating SWD’s role in hepatic steatosis in MASLD patients, in a prospective study comparing 2D-SWE and histological findings. SWD identified lobular inflammation, securing an AUC of 0.95 for minor inflammation. Nonetheless, AC was superior to SWD in evaluating steatosis, suggesting that SWD could be more effective for inflammation rather than steatosis [68] (Table 1).

Da Silva showed that SWE and SWD are highly sensitive and specific in quantifying hepatic steatosis when compared to MRI PDFF, highlighting their potential as non-invasive tools for liver steatosis assessment. This retrospective study included 15 patients undergoing both ultrasound (SWE, SWD, and ATI) and MRI for liver disease. There is a strong correlation between SWD and PDFF, suggesting that SWD could identify hepatic steatosis and measure liver fat without invasive methods [69].

Studies in patients with alcohol-related steatosis are limited, however some conclusions can be drawn from MASLD, where SWD is part of MPUS. Non-invasive methods have been used to assess MASLD severity and hepatic steatosis. PDFF via MRI is the gold standard for liver fat quantification in MASLD [70,71] (Table 1). Multiparametric MRI with PDFF measurement is a promising tool for fat content, iron load, and fibrosis quantification in MASLD [72]. Moreover, imaging studies showed that SWD values rise with steatosis severity, likely due to changes in the liver’s viscoelastic properties [51]. ALD and MASLD are two different medical conditions that cause different steatosis patterns that affect how LS measurements are interpreted with SWD [73]. Microvesicular steatosis, higher inflammation, and fibrosis levels can change the acoustic properties of the liver in ALD, which can lead to different SWD cut-off values than in MASLD, where macrovesicular steatosis is more common [74].

Establishing ALD-specific cut-off values is imperative, requiring studies similar to MASLD studies. They should compare SWD values to MR-tomography PDFF and find correlations with qualitative and quantitative histological findings. This could help us understand how ALD steatosis impacts the body and develop non-invasive ALD diagnosis methods.

### 4.2. Inflammation

SWD emerged as a non-invasive tool for assessing necroinflammation in liver tissue, providing information supplementary to traditional measures of fibrosis.

Zhang et al. (2022) observed that SWDS values increased with necroinflammatory grade, from 12.3 (10.6–14.5) (m/s)/kHz in grade A0 to 16.6 (13.4–17.5) in grade A3 [40]. Furthermore, Sun et al. (2022) highlighted that SWDS values were significantly higher in patients with variceal hemorrhage (17.0 ± 1.45) compared to those without (15.2 ± 1.88), underlining its potential role in assessing portal hypertension complications [11].

Moreover, due to reduced immune function and gut bacterial translocation, ALD patients are at risk of infections and sepsis. This elevated sepsis risk can exacerbate necroinflammation, further complicating ALD therapy and prognosis.

An important aspect of SWD’s diagnostic capabilities is how liver tissue microstructure affects shear wave speed measurements.

Preclinical studies showed that the SWD slope significantly increased earlier than the shear wave speed and correlated better with pathological severity, indicating a tight relationship between SWD and microstructure [75].

This was confirmed by Lee et al. (2019) in liver transplant recipients. Allograft injury increased liver stiffness and SWD values. The latter was better at diagnosing necroinflammatory activity than hepatic stiffness alone [12]. Some investigations have found SWD to be an independent factor in liver necroinflammatory activity, revealing tissue alterations beyond fibrosis [76]. Research also confirmed a significant positive correlation in SWD values between grades of inflammation in pediatric patients with liver disease [77].

In AATD, a study by Shulz et al. aimed to evaluate the role of SWD in assessing liver inflammation, finding a strong correlation between SWD values and liver inflammation grades, with higher values indicating more severe inflammation. These results underscore SWD’s capability to non-invasively assess liver disease severity across different patient groups [66].

#### Inflammation in MASLD

As with MASH, MASLD inflammatory quantification is difficult for non-invasive diagnosis. Jang et al. examined SWD’s ability to assess hepatic inflammation in 132 MASH patients in a multicenter, cross-sectional study. Patients had MPUS, with biopsy as the gold standard. The findings revealed that DS values had a notable correlation with lobular inflammatory activity, inferring that SWD is efficient in evaluating liver inflammation and fibrosis, thus furnishing a non-invasive diagnostic approach for MASH [77,78] (Table 1). Another study found that a risk score algorithm, incorporating AC from ATI and DS from 2D-SWE, was better at diagnosing than serum markers or US parameters used by themselves. Sugimoto et al. investigated SWD’s capability in detecting hepatic inflammation to identify high-risk MASH. This retrospective cross-sectional study, involving 111 Japanese and 102 Korean patients with biopsy-confirmed MASLD, using MPUS, showed a significant association between DS and lobular inflammation and accurately found high-risk MASH [68]. A prospective study of Gao et al. encompassed 21 adults, grouped into normal and steatotic liver groups based on MRI-PDFF, to evaluate SWD’s role in hepatic inflammation. The research used MPUS to capture the SWV, SWD, ATI, NLV, and L/K ratio. The SWV, SWD, ATI, NLV, L/K ratio, and MRI-PDFF differed significantly between normal and steatotic livers. This showed that SWD can detect hepatic inflammation [79]. These findings indicated that MPUS, particularly DS, may be a promising non-invasive liver inflammatory evaluation method in MASLD. Comparing SWD and MASLD studies on ALD inflammation revealed similarities and differences. MASH-related liver inflammation can be detected by MPUS parameters like DS, AC, and shear wave speed through 2D SWE [51]. Liver inflammation drives ALD and MASLD progression and DS has been linked to histological evaluations of lobular inflammation, a key MASH feature [12,51,80]. Pro-inflammatory cytokines and ROS are released in ALD because of the toxic effects of ethanol metabolism. This is similar to MASLD’s inflammatory cascade, but it happens through different metabolic processes [81,82].

Utilizing MASLD study-derived insights, it can be posited that ALD may exhibit analogous SWD parameter shifts correlating with the degree of inflammation. Nonetheless, distinct etiology, such as inflammation induced by acetaldehyde in ALD versus inflammation precipitated by metabolic syndrome in MASLD, could affect the liver’s viscoelastic properties in divergent manners. Hence, while direct extrapolation is not wholly viable owing to these varied pathophysiological pathways, utilizing 2D SWE parameters to detect and gauge inflammation severity in the liver seems plausible. Thus, assessing the DS, AC, and shear wave speed in ALD might remain a beneficial non-invasive instrument for evaluating and keeping track of liver inflammation. Prospective studies with biopsy-confirmed ALD patients employing these multiparametric US techniques could yield valuable insights into the efficacy of SWD in this context. This could, in turn, enhance the comprehension of ALD inflammation and contribute to the development of non-invasive diagnostic and monitoring methodologies for this cohort.

### 4.3. Fibrosis

SWD has undergone extensive examination for the evaluation of liver fibrosis, showing its ability to assess the elastic and viscous properties of liver tissue, thus providing a comprehensive assessment of liver health.

The progressive increase in SWDS values with fibrosis severity has been documented. Ferraioli et al. (2021) reported mean SWDS values of 9.8 [8.8–10.8] (m/s)/kHz for F0–1, 13.6 [12–14.8] for F2, and 17.5 [13.7–20.5] for F3–4 [60]. Similarly, Wang et al. (2022) demonstrated increases from F0 (11.8 ± 0.39) to F4 (17.4 ± 0.34) in patients undergoing hepatectomy, confirming SWD’s utility in fibrosis staging [51].

SWD has demonstrated high diagnostic efficacy in assessing both inflammation and fibrosis, with shear wave speed more accurately indicates fibrosis, whereas a dispersion slope is more reflective of necroinflammation [9]. A computational modeling study found significant variability in shear wave speed measurements in liver fibrosis, up to 56%, due to elastic wave scattering and frequency-dependent attenuation, highlighting the need for further research on microstructural effects in SWE [83].

Wang et al. (2022) compared SWE and SWD for the identification hepatic fibrosis in 210 HCC patients scheduled for hepatectomy. SWE demonstrated better ROC AUC values than SWD in predicting severe fibrosis and cirrhosis, despite their significant correlation with histological fibrosis stages [51]. According to Seyerek et al., SWD was effective in detecting necroinflammation, while 2D-SWE and TE were essential for detecting hepatic fibrosis [84]. In pediatric liver fibrosis, Cetinic et al. found that moderate to severe inflammation raised SWD values, indicating liver viscosity [56].

Studies revealed a positive association between SWD values and fibrosis grade, suggesting that as fibrosis advances, SWD values rise due to alterations in LS and viscosity. Ferraioli et al. affirmed 2D-SWE’s accuracy in fibrosis staging, correlating SWD highly with liver fibrosis but not with steatosis [65], while Deffieux et al. demonstrated that SWD via supersonic shear imaging (SSI) had comparable accuracy to FibroScan for fibrosis staging [59]. Cetinic et al. (2023) studied SWD’s fibrosis evaluation role in pediatric liver transplant patients [77].

A sum of 48 pediatric recipients of liver transplants were subjected to 2D-SWE and SWD for the assessment of liver stiffness (LS) at times of follow-up biopsies. The SWD was found to be related with liver fibrosis and inflammation, suggesting it as a non-invasive biomarker for liver graft monitoring [77]. Nagasawa et al. showed that the SWD dispersion slope detects significant fibrosis in Fontan-associated liver disease, with strong correlations to central venous pressure and liver stiffness [85].

#### Fibrosis in MASLD

Popa et al. examined how shear wave dispersion (SWD) affected liver fibrosis in 215 MASLD patients. A cross-sectional study used multiparametric ultrasound (MPUS) methods like Vi.PLUS to assess liver fibrosis, steatosis, and viscosity. In patients with severe fibrosis, SWD viscosity increased. Vi.PLUS values were independently related to liver stiffness (LS) measurements and body mass index, indicating that SWD is a non-invasive tool for assessing liver fibrosis in MASLD patients, reflecting hepatic damage and inflammation [67].

Sugimoto et al. examined the diagnostic performance of three ultrasound markers—the liver stiffness (LS), attenuation coefficient (AC), and dispersion slope (DS)—for non-invasive liver fibrosis, steatosis, and inflammation assessment in MASLD. This cross-sectional study included 111 Japanese and 102 Korean MASLD patients undergoing liver biopsy and 2D-SWE. All the US markers accurately identified high-risk MASH with significant fibrosis, demonstrating the clinical utility of these non-invasive ultrasonography markers in MASLD patients [68].

Table 1 lists the main studies addressing the assessment of liver fibrosis and steatosis using elastography.

**Table 1:** Summary of key studies evaluating ultrasound techniques for assessing liver fibrosis and steatosis.

Author, Year	Study Design	Main Results
Popa et al., 2021 [84]	Cross-sectional study	SSp.PLUS correlated better with CAP values compared to Att.PLUS. In patients with severe fibrosis, SWD viscosity increased. Vi.PLUS values were independently related to liver stiffness (LS) measurements and body mass index, indicating that SWD is a non-invasive tool for assessing liver fibrosis in MASLD patients, reflecting hepatic damage and inflammation.
Sugimoto et al., 2021 [64]	Cross-sectional study	SWD identified lobular inflammation, securing an AUC of 0.95 for minor inflammation. AC was superior to SWD in evaluating steatosis.
Da Silva et al., 2023 [65]	Retrospective study	SWE and SWD are highly sensitive and specific in quantifying hepatic steatosis when compared to MRI PDFF.
Idilman et al., 2013 [67]	Retrospective study	PDFF was effective in differentiating moderate or severe hepatic steatosis from mild or no hepatic steatosis.
Jang et al., 2022 [74]	Cross-sectional study	SWD is efficient in evaluating liver inflammation and fibrosis.
Gao et al., 2021 [12]	Prospective study	The SWV, SWD, ATI, NLV, L/K ratio, and MRI-PDFF differed significantly between normal and steatotic livers. SWD can detect hepatic inflammation.
Sugimoto et al., 2020 [82]	Cross-sectional study	The combination of dispersion slope, attenuation coefficient, and shear wave speed measurements showed good diagnostic performance for the diagnosis of NASH.
Ferraioli et al., 2020 [61]	Cross-sectional study	The 2D-SWE technique showed a high correlation with TE values for liver stiffness measurement, identifying significant or severe fibrosis.

Abbreviations: 2D-SWE, Two-Dimensional Shear Wave Elastography; AC, Attenuation Coefficient; AUC, Area Under the Curve; ATI, Attenuation Imaging; Att.PLUS, Attenuation Plus; CAP, Controlled Attenuation Parameter; L/K Ratio, Liver-to-Kidney Ratio; LS, Liver Stiffness; MASLD, Metabolic Dysfunction-Associated Steatotic Liver Disease; MRI-PDFF, Magnetic Resonance Imaging Proton Density Fat Fraction; NASH, Non-Alcoholic Steatohepatitis; NLV, Normalized Liver Volume; PDFF, Proton Density Fat Fraction; SSp.PLUS, Shear Wave Dispersion Plus; SWD, Shear Wave Dispersion; SWE, Shear Wave Elastography; SWV, Shear Wave Velocity; TE, Transient Elastography; Vi.PLUS, Viscosity Plus.

The non-invasive imaging approach 2D-SWE strongly correlated with liver stiffness and fibrosis phases in 210 patients receiving hepatectomy for hepatocellular cancer in a prospective study. A higher viscosity indicated advanced fibrosis. Other imaging parameters outperformed SWD, which confirmed a correlation with hepatic inflammation. Accordingly, SWD may be a potential non-invasive liver fibrosis assessment instrument that could reduce liver biopsies and improve disease progression staging [51].

Research by Sugimoto et al. showed that 2D-SWE could accurately assess liver inflammation, steatosis, and fibrosis in 120 adults with MASLD who had a liver biopsy. The main ultrasound parameters measured were the DS, AC, and shear wave speed, where DS indicated lobular inflammation, AC revealed steatosis, and shear wave speed detected fibrosis [86] (Table 1). The significance of these non-invasive tests for a comprehensive MASLD evaluation is thus underscored. Collectively, these studies highlight the efficiency of shear wave-based methodologies in the non-invasive assessment of liver fibrosis in MASLD [11].

Analyzing SWD in ALD fibrosis can parallel MASLD research. The existing literature confirms that SWD, including 2D-SWE, closely matches liver fibrosis phases, often validated by liver biopsy. Liver fibrosis predicts ALD and MASLD mortality and disease decompensation [35]. This emphasizes the clinical value of non-invasive fibrosis testing and staging approaches.

SWD can measure liver fibrosis and inflammation in MASLD without invasive procedures. 2D-SWE, including ShearWave PLUS, can evaluate fibrosis with equal success to TE [60]. SWD includes liver tissue’s elasticity and viscosity, providing molecular information on microstructural alterations [48].

Considering the natural history of ALD, using SWD could also provide similar benefits in diagnosing and staging fibrosis in these patients. The ShearWave PLUS stability index (SI) tool has the potential to greatly improve the reliability of liver stiffness measurements (LSMs) and reduce variability. Its effectiveness in evaluating ALD will depend on how well it correlates with histological fibrosis scores, which has shown promising results in patients with MASLD [68,87] (Table 1).

SWD discrepancies in ALD versus MASLD across diverse fibrosis stages might be modulated by several factors, such as differing liver disease etiologies, fibrogenesis patterns, and unique histopathological variations [68]. SWD values in ALD might present distinct cut-off points or correlations with fibrosis stages when compared to MASLD. Liver viscoelastic properties, assessed via SWE, bear the influence of both fibrotic collagen and tissue inflammation. Thus, for ALD, bespoke reference values or SWE measurement interpretative algorithms might be requisite to accommodating the differing histopathological landscapes [12]. The molecular differences between ALD and MASLD suggest that SWD may be useful in LS and fibrosis assessments, but it must be calibrated and validated against ALD histological benchmarks. More comparative research is needed. SWD measurement from ALD and MASLD cohorts, confirmed with histology, is crucial to fine-tuning SWD methods and proving their diagnostic efficacy in ALD. Using sophisticated tools like the Stiffness Index (SI) may improve SWD measurements and ALD fibrotic evaluations.

## 5. Conclusions

SWD imaging is a promising option for the diagnosis and monitoring of ALD. It has the ability to capture molecular and histopathological subtleties, providing personalized and precise diagnostic and therapeutic methods. Due to a lack of ALD studies, SWD struggles to achieve specificity.

The histological and molecular characteristics of ALD limit the direct translation of MASLD knowledge. In personalized medicine, incorporating SWD into monitoring procedures becomes relevant for comprehensive disease examination. Solid clinical validations are required to determine the relationship between SWD and particular histological abnormalities in ALD. New techniques and technologies are needed to boost the diagnostic and prognostic abilities of SWD in ALD. Large-scale prospective studies are necessary to confirm SWD’s role as a prognostic tool in ALD.

## Data Availability

Not applicable.
